# The effect of small-sided games and HIIT formats and competitive level on enjoyment and exercise intensity in young adult male soccer players

**DOI:** 10.5114/biolsport.2025.150046

**Published:** 2025-05-08

**Authors:** Weiqiang Xu, Robert Trybulski, Rui Miguel Silva, Yichen Zhao, Henrique de Oliveira Castro, Gustavo De Conti Teixeira Costa, Filipe Manuel Clemente

**Affiliations:** 1 Gdansk University of Physical Education and Sport, 80-336 Gdansk, Poland; 2 Medical Department Wojciech Korfanty, Upper Silesian Academy, Katowice, Poland; 3 Department of Physical Therapy and Ergotherapy, Ivan Boberkyj Lviv State University of Physical Culture, 79007 Lviv, Ukraine; 4 Escola Superior Desporto e Lazer, Instituto Politécnico de Viana do Castelo, Rua Escola Industrial e Comercial de Nun’Álvares, 4900-347 Viana do Castelo, Portugal; 5 Sport Physical Activity and Health Research & Innovation Center, Viana do Castelo, Portugal; 6 Department of Physical Education, Xiamen University, 361005 Xiamen, China; 7 Grupo de Estudos e Pesquisas em Educação Física e Esportes, Laboratório de Análise Esportiva, Universidade Federal de Mato Grosso, Cuiabá, 78060-900, Brazil; 8 Núcleo de Estudo e Pesquisa Avançada em Esportes, Universidade Federal de Goiás, Goiânia 74690-900, Brazil

**Keywords:** Football, Conditioned games, High-intensity interval training, Contentment, Physical fitness

## Abstract

This study aims to analyze the differences in Physical Activity Enjoyment Scale (PACES) scores and exercise intensity across small-sided games (SSGs) and high-intensity interval training (HIIT) formats among soccer players from Tier 2 and Tier 3 competitive levels. Utilizing a cross-sectional design, 77 male players (20.64 ± 1.56 years) participated in four training sessions under controlled conditions. Enjoyment was assessed with PACES, while exercise intensity was monitored through heart rate and rating of perceived exertion (RPE). The training formats included 1 v 1 and 5 v 5 SSGs and long and short HIIT formats. Players were categorized as Amateur (Tier 2) or Competitor (Tier 3) based on training volume and performance level. The training formats included 1 v 1 and 5 v 5 SSGs and long and short HIIT formats. The present study revealed significant differences in PACES scores across the different training formats (p < 0.001), with the 5 v 5 format yielding the highest scores and long HIIT the lowest. A significant interaction between training format and competitive level was observed (p = 0.011). Tier 2 players reported higher PACES scores in the 5 v 5, 1 v 1, and Short HIIT formats compared to Tier 3 players. SSGs and HIIT formats significantly influence enjoyment, with smaller-sided formats (1 v 1) and short HIIT showing the highest enjoyment levels. Tier 2 players reported higher enjoyment and physical efforts than Tier 3 players, in 5 v 5, 1 v 1, and short HIIT formats. These findings show the importance of programming training sessions according to the competitive level of athletes to maximize enjoyment and manage effort in soccer training programs.

## INTRODUCTION

Soccer is a high-intensity, intermittent sport that typically lasts 90 minutes or more, with performance depending on players’ technical, tactical, physiological, and psychological characteristics. During a match, players engage in activities ranging from standing still to maximal running, with intensity fluctuating and involving short sprints, physical duels, and sudden changes of direction, interspersed with partial recovery at low intensity [1, 2]. Because of these demands, soccer requires players to perform prolonged intermittent exercise, high-intensity efforts, and sprints and produce high-power outputs in actions such as kicking, jumping, and tackling [3]. To meet these diverse physical demands, multiple training formats are employed in soccer, including High-Intensity Interval Training (HIIT) and Small-Sided Games (SSGs) [4, 5]. Adopting different intensities and formats can comprehensively improve aerobic fitness, sprint performance, change of direction, technical skills, and tactical behaviors, while also maintaining player engagement and motivation, ultimately enhancing overall training effectiveness [6, 7].

HIIT is a training modality alternating short bouts of high-intensity effort with recovery periods [8]. It is defined as repeated short (< 45 s) to long (2–4 min) bouts of relatively high, but not maximal-intensity, exercise [9]. HIIT can improve fitness variables such as maximal oxygen uptake [10], repeated sprint ability [11], and sprinting capacity [12]. SSGs, also known as small-sided or conditioned games, are game-based drills played on smaller pitches with modified rules and fewer players, which alter the structural dynamics of a formal match [13, 14]. Despite being more skill-based, SSGs can be considered a form of HIIT because they can induce high physiological loads by adjusting pitch dimensions and conditions [2]. The main difference between SSGs and traditional running-based HIIT is the presence of the ball, the consistent use of SSGs may further improve specific technical skills and tactical behaviors [14, 15].

In professional soccer players, both HIIT and SSGs sessions induce similar physiological responses, however, HIIT produces a mood disturbance while SSGs maintain mood balance [16]. Variations in the structure of SSGs, such as format, pitch configuration, and training regimen, lead to differing outcomes [17, 18]. For instance, larger pitch sizes significantly affect internal load responses (Heart Rate – HR and Rating of Perceived Exertion – RPE), increase total and high-speed running distances, and promote greater player dispersion at a collective level [19]. Additional research is needed to clarify how these structural variations interact with psychological factors and inter- and intra-individual variability [20].

Enjoyment, an individual’s positive affective response to an activity, is fundamental to sustaining motivation and long-term adherence [21, 22]. It reflects how enjoyable and intrinsically rewarding the activity is, making it a key factor in sports commitment [23]. The Physical Activity Enjoyment Scale (PACES) is commonly used to measure this construct in various exercise contexts, including soccer training. PACES has shown acceptable internal consistency and test-retest reliability [21], and its widespread use underscores the importance of enjoyment in soccer contexts [24, 25]. Higher enjoyment can facilitate greater work output and elicit stronger physiological responses [26, 27]. Research on enjoyment in soccer training indicates that higher enjoyment is associated with positive psychometric responses (e.g., emotional balance, motivation) [28, 29], which can improve players’ physical performance and engagement during training [29, 30].

The balance between physical fitness development and enjoyment is a key concern for coaches and athletes in daily training. Studies have shown that SSGs are often more enjoyable than HIIT of similar exercise intensity in soccer players [7], and lead to higher PACES scores while maintaining aerobic fitness [5]. Moreover, enjoyment levels differ across SSGs formats (e.g., comparing 3 vs. 3 and 4 vs. 4) [31]. Regarding competitive levels, elite athletes generally report higher sports enjoyment [32], although the definition of “elite” varies widely. Consequently, the 6-tiered Participant Classification Framework [33] can help clarify distinctions, such as those between Tier 2 (Trained/Developmental) and Tier 3 (Highly Trained/National Level).

Despite these insights, most existing research has focused on comparing a single SSGs format with one type of HIIT or examining only one competitive level. This narrow scope makes it unclear whether enjoyment differs across multiple training formats (e.g., different SSGs formats or different HIIT durations) or multiple competitive levels. Furthermore, the effects of SSGs (e.g., 1 v 1) compared with medium-sided games (MSGs, e.g., 5 v 5), as well as long versus short HIIT intervals, remain understudied. Addressing these gaps could optimize both the physical and psychological aspects of training.

Therefore, this study aimed to evaluate the differences in PACES scores and exercise intensity across distinct SSGs and HIIT formats in soccer players from Tier 2 and Tier 3 competitive levels. It is hypothesized that soccer players at different competitive levels will exhibit significant differences in physical activity enjoyment across these training formats, with higher-level athletes (Tier 3) expected to report greater enjoyment due to their advanced fitness levels and familiarity with structured training, compared to Tier 2 athletes.

## MATERIALS AND METHODS

### Study design

A cross-sectional design was employed in this study to examine the influence of training formats: 5 v 5 (MSGs), 1 v 1 (SSGs), long HIIT, and short HIIT; and competitive levels: Tier 2 and Tier, aligned with the Participant Classification Framework proposed by McKay et al. (2022) [33]on physical activity enjoyment in soccer players. Using convenience sampling, participants were recruited from four teams in the China University Football Association (CUFA). Two teams were Tier 2, and the other two were Tier 3. Each team consisted of 20 players, resulting in a total sample of 80 players. All players were male soccer players aged 18–24 years, actively competing in CUFA. Players were excluded if they could not complete all training sessions or assessments.

Due to logistical constraints, experiments for the four teams were conducted at different times, but all followed a pre-determined order of assessments and training sessions over two weeks. Each team participated in one fitness assessment and four training sessions. The fitness assessment is a 30–15 intermittent fitness test (30–15_IFT_). Each session performed a different training format, which included 5 v 5 MSGs and 1 v 1 SSGs, and long and short HIIT. To ensure consistency in the experimental conditions, assessment and training sessions were conducted on an outdoor artificial grass pitch at the same time each day (16:00–18:00) and under similar weather conditions. This approach was employed to minimize the potential influence of environmental factors on performance and enjoyment.

An online randomization tool (www.randomizer.org) was used to allocate players within each team into two groups: Group A (n = 10) and Group B (n = 10). The order of the training forms is different for Groups A and B. Details of the study setting are provided in Table 1. Before each training session, players performed the FIFA 11+ standardized warm-up (Level 3). During the training, players’ HRs were monitored using an optical heart rate monitor (INSAIT KS System, GenGee, China). After each training session, players completed the RPE and PACES to evaluate their subjective training exertion and enjoyment level.

**TABLE 1:** Study setting

Team	Group	Session 1	Session 2	Session 3	Session 4
**Team 1 (Tier 3)**	Group A	5 v 5 MSGs	Long HIIT	1 v 1 SSGs	Short HIIT

	Group B	Long HIIT	5 v 5 MSGs	Short HIIT	1 v 1 SSGs

**Team 2 (Tier 2)**	Group A	5 v 5 MSGs	Long HIIT	1 v 1 SSGs	Short HIIT

	Group B	Long HIIT	5 v 5 MSGs	Short HIIT	1 v 1 SSGs

**Team 3 (Tier 3)**	Group A	1 v 1 SSGs	Short HIIT	5 v 5 MSGs	Long HIIT

	Group B	Short HIIT	1 v 1 SSGs	Long HIIT	5 v 5 MSGs

**Team 4 (Tier 2)**	Group A	1 v 1 SSGs	Short HIIT	5 v 5 SSGs	Long HIIT

	Group B	Short HIIT	1 v 1 SSGs	Long HIIT	5 v 5 MSGs

Note: SSGs = Small-Sided Games; HIIT = High-Intensity Interval Training; MSGs = Medium Sided-Games

### Participants

The sample size was determined using G*Power software (version 3.1.9.7) for a repeated-measures ANOVA with a within-between interaction. The calculation was based on a medium effect size (f = .25), a significance level of α = .05, a statistical power of 1−β = .80, four measurements (training formats), two groups (competitive levels), a correlation among repeated measures of 0.5, and a nonsphericity correction factor (ε = .75). The required total sample size was 30 participants. To account for potential dropouts, 80 male soccer players from the CUFA were initially recruited. Of these, three players were excluded for failing to attend all sessions, resulting in a final sample of 77 participants. This exceeded the minimum requirement, ensuring adequate statistical power for detecting significant effects. Details on ages, height, weight, BMI, and V_IFT_ are presented in Table 2.

**TABLE 2:** Characteristics of participants.

Characteristic	Competitive levels		

	Tier 2 (N = 37)	Tier 3 (N = 40)	All (N = 77)
**Age (years)**	20.35 ± 1.34	20.90 ± 1.72	20.64 ± 1.56
**Height (cm)**	178.49 ± 4.85	180.78 ± 5.50	179.68 ± 5.29
**Weight (kg)**	70.19 ± 6.29	73.15 ± 6.49	71.73 ± 6.52
**BMI**	22.04 ± 1.89	22.36 ± 1.34	22.20 ± 1.62
**V_IFT_ (km/h)**	19.22 ± 1.04	20.03 ± 1.68	19.64 ± 1.46

Convenience sampling was used to recruit teams that met the inclusion criteria of actively competing in CUFA and having a minimum of 20 available players. Two teams are from CUFA Super Champions League (CUFA Division 1) and CUFA League One (CUFA Division 2), which are national-level competitions. Players engage in structured and periodized training close to the maximum training volume of soccer. The other two are the CUFA Provincial League (CUFA Division 4) teams, a regional-level competition. Players train regularly, at least three times a week. Based on the participant classification framework into six levels (Tier 0~5) [34], CUFA Division 1 and 2 players are classified as Tier 3 (highly trained/national level), and CUFA Division 4 players are classified as Tier 2 (trained/developmental). The inclusion criteria included: (i) players participating in the same training session are from the same team; (ii) no injury or illness reported one month before the study and during the study; (iii) no physical or cognitive disease reported; (iv) no drugs reported; (v) no alcohol reported one month before the study. The exclusion criteria included: (i) no regular presence of participants in training sessions; (ii) participants fell ill or injured during the study period.

The principles of the Declaration of Helsinki conducted this study. Ethical approval was granted by the Medical Ethics Committee of Xiamen University (Approval Number: XDYX202406K34). The trial was prospectively registered in the Chinese Clinical Trial Registry (Registration Number: ChiCTR2400087494). Written informed consent was obtained from all participants before the experiment, with participation being entirely voluntary.

### Anthropometric measurement

Players were instructed to remove their shoes and wear a t-shirt and shorts for the height and weight measurements, which were recorded in centimeters (cm) and kilograms (kg) using a calibrated SECA 213 stadiometer (Seca GmbH & Co. KG, Hamburg, Germany) and a Tanita BC-558 digital scale (Tanita Corporation, Tokyo, Japan). Following these measurements, players rested in a supine position on a bed for 10 minutes, during which the minimum HR recorded was taken as the resting HR. Standardized forms were used to document basic demographic and physiological information, including age, height, weight, and resting HR.

### Cardiorespiratory assessment

Players were instructed to avoid strenuous exercise for 48 hours before the fitness assessment to ensure accurate results. The assessment was performed from 16:00 to 18:00 outdoors on artificial grass, with an average temperature of 22 ± 2.45°C and humidity of 54 ± 19.06%. Before the test, players completed a FIFA 11+ standardized warm-up (Level 3). Following the warm-up, a 3-minute recovery period was provided to ensure readiness for the test. The fitness test was the 30–15 Intermittent Fitness Test (30–15_IFT_). The 30–15_IFT_ consists of 30-second shuttle runs at progressively increasing speeds, interspersed with 15-second recovery periods, where players shuttle between 40-meter lines, guided by audio beeps until they fail to maintain the required speed for three consecutive attempts, with the final stage’s velocity recorded as V_IFT_ [35]. This assessment was conducted only for HIIT programming purposes and was not to be considered for analysis, as this was beyond the scope of the present study.

### SSGs Intervention

After completing the standardized FIFA 11+ warm-up (Level 3), players rested for a 3-minute recovery period before participating in SSGs on an outdoor artificial pitch, with each player allocated an area of 175 m^2^, a dimension chosen to ensure optimal player involvement, decision-making opportunities, and movement intensity, as used in previous research on small-sided games [36]. Two formats of SSGs were implemented: 5 v 5 (MSGs) and 1 v 1 (SSGs). The 5 v 5 + Goalkeeper format was conducted on a 50 × 35-meter pitch with seven-a-side goals. Each session lasted 25 minutes, divided into four 4-minute sets with 3 minutes of passive recovery between sets. The 1 v 1 + Goalkeeper format was played on a 25 × 14-meter pitch with five-a-side goals. These sessions also lasted 25 minutes but consisted of eight 1-minute sets, with 2 minutes of passive recovery between sets, except for a longer 5-minute recovery period between the 4^th^ and 5^th^ sets. Goalkeepers were excluded from the study sample and were not analyzed due to their distinct training requirements. The offside rule was not applied. Players were instructed to exert maximum effort and maintain ball possession for as long as possible. Two coaches positioned around the pitch provided new balls whenever necessary to ensure continuous play.

### HIIT Intervention

Before each HIIT session, players completed a standardized FIFA 11+ warm-up (Level 3), followed by a 3-minute recovery period. The HIIT sessions took place on an outdoor artificial grass pitch and were implemented in two distinct formats. The Long HIIT format lasted 25 minutes and consisted of four 4-minute work periods performed at 80% of V_IFT_, with 3 minutes of passive recovery between sets. In contrast, the Short HIIT format also lasted 25 minutes but comprised eight 1-minute work periods performed at 95% of V_IFT_, with 2 minutes of passive recovery between sets, except for a longer 5-minute recovery period between the 4^th^ and 5^th^ sets.

### Exercise Intensity Quantification

Exercise intensity was objectively assessed by continuously recording their HR during each training session, excluding the warm-up, using an optical heart rate monitor (INSAIT KS System, GenGee, China). HR data were analyzed from the start to the end of each HIIT and SSGs session, with the mean HR (HRmean) calculated to represent the average exercise intensity for each session. Additionally, RPE rating was recorded immediately after each session using the Borg CR10 scale [37], a validated tool for reliably estimating effort intensity in athletic populations. Players were instructed to rate their perceived effort individually to avoid influence from their peers’ scores. While subjective, the RPE scale provides valuable complementary insights into exercise intensity when combined with objective measures.

### PACES

Five minutes after each session, players completed the PACES to evaluate their enjoyment of the activity. They were instructed to rate their current feelings about the physical activity they had just performed. The PACES inventory consists of 18 items rated on a 7-point bipolar scale, with 11 items reverse-scored. The total enjoyment score is obtained by summing the ratings for all items, yielding a possible range of 18 to 126. Higher PACES scores reflect greater levels of enjoyment in physical activity [21].

### Statistical Analysis

All analyses were conducted using SPSS (version 26.0, IBM). Descriptive statistics were presented as mean ± standard deviation. Potential outliers were identified through boxplots, defined as values exceeding 1.5 times the interquartile range from the box edges. Normality was assessed using the Kolmogorov-Smirnov test, and Normal Q-Q plots were visually inspected. Levene’s test was used to confirm the homogeneity of variances. A two-way mixed ANOVA was performed to evaluate the effects of training format (withinsubject factor) and competitive level (between-subject factor) on physical activity enjoyment. Post-hoc comparisons for main effects were adjusted using Bonferroni corrections. When a significant interaction was identified, simple main effects were analyzed using separate one-way ANOVAs and repeated-measures ANOVAs, focusing on differences between competitive levels within each training format and differences between training formats within each competitive level. Effect sizes were reported as partial eta squared (ηp^2^) to quantify the magnitude of significant effects, with values of 0.01, 0.06, and 0.14 interpreted as small, medium, and large effects, respectively [38]. Statistical significance was set at p < .05.

## RESULTS

There were three outliers in the data, as assessed by inspection of a boxplot. The two-way mixed ANOVA was conducted both with and without these outliers. As the inclusion or exclusion of these outliers did not alter the statistical significance of the findings, they were retained in the final analysis. Although the Kolmogorov-Smirnov test indicated that some cells deviated from normality, the Normal Q-Q plots suggested that the data distribution was approximately normal. Levene’s test indicated homogeneity of variances (p > .05). However, Box’s test showed that the assumption of homogeneous covariances was not met (p < .001). Mauchly’s test of sphericity indicated that the assumption of sphericity was met for the two-way interaction [χ^2^(5) = 8.23, p = .144]. Table 3 describes the PACES scores for the different competitive levels and training formats. A statistically significant interaction between competitive level and training format on PACES score was found [F (3,225) = 3.83, p = .011, $\eta_{\text{p}}^{2}$ = .049].

**TABLE 3:** PACES scores for different competitive levels and training formats.

Training formats	Competitive levels		Two-way mixed ANOVA

	Tier 2 (N = 37)	Tier 3 (N = 40)	
**5 v 5 MSGs**	97.84 ± 17.29	86.40 ± 19.12	time × group
**1 v 1 SSGs**	93.32 ± 19.18	92.13 ± 17.96	F (3,225) = 3.83
**Long HIIT**	85.22 ± 21.53	74.05 ± 19.17	p = .011
**Short HIIT**	91.68 ± 19.93	75.33 ± 22.98	$\eta_{\text{p}}^{2}$ = .049

Note: SSGs = Small-Sided Games; HIIT = High-Intensity Interval Training; MSGs = Medium Sided-Games

**FIG. 1:**
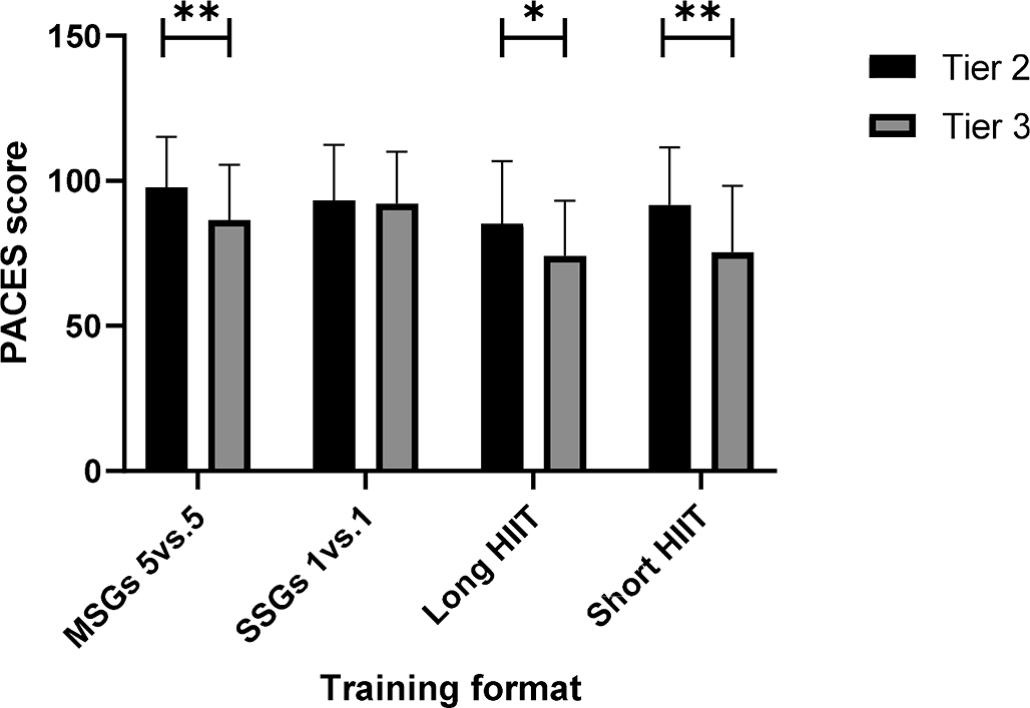
PACES score with simple main effect for differences between competitive levels within each training format. *Denotes significance at p < 0.05; **Denotes significance at p < 0.01. Note: SSGs = Small-Sided Games; HIIT = High-Intensity Interval Training; MSGs = Medium Sided-Games

Figure 1 illustrates the simple main effects of differences between competitive levels within each training format. At 5 v 5 MSGs, there was a statistically significant difference in PACES score between competitive levels [F (1, 75) = 7.54, p = .008, $\eta_{\text{p}}^{2}$ = .091]. Tier 2 had significantly higher PACES scores than Tier 3 (M difference = 11.44, SE = 4.17, p = .008). At 1 v 1 SSGs, no statistically significant difference emerged [F (1, 75) = 0.08, p = .778, $\eta_{\text{p}}^{2}$ = .001]. Tier 2 did not differ significantly from Tier 3 (M difference = 1.20, SE = 4.23, p = .778). There was a statistically significant difference at long HIIT [F (1, 75) = 5.79, p = .019, $\eta_{\text{p}}^{2}$ = .072]. Tier 2 had significantly higher PACES scores than Tier 3 (M difference = 11.17, SE = 4.64, p = .019). A statistically significant difference was also found at short HIIT [F (1, 75) = 11.04, p = .001, $\eta_{\text{p}}^{2}$ = .128]. Tier 2 had significantly higher PACES scores than Tier 3 (M difference = 16.35, SE = 4.92, p = .001).

**FIG. 2:**
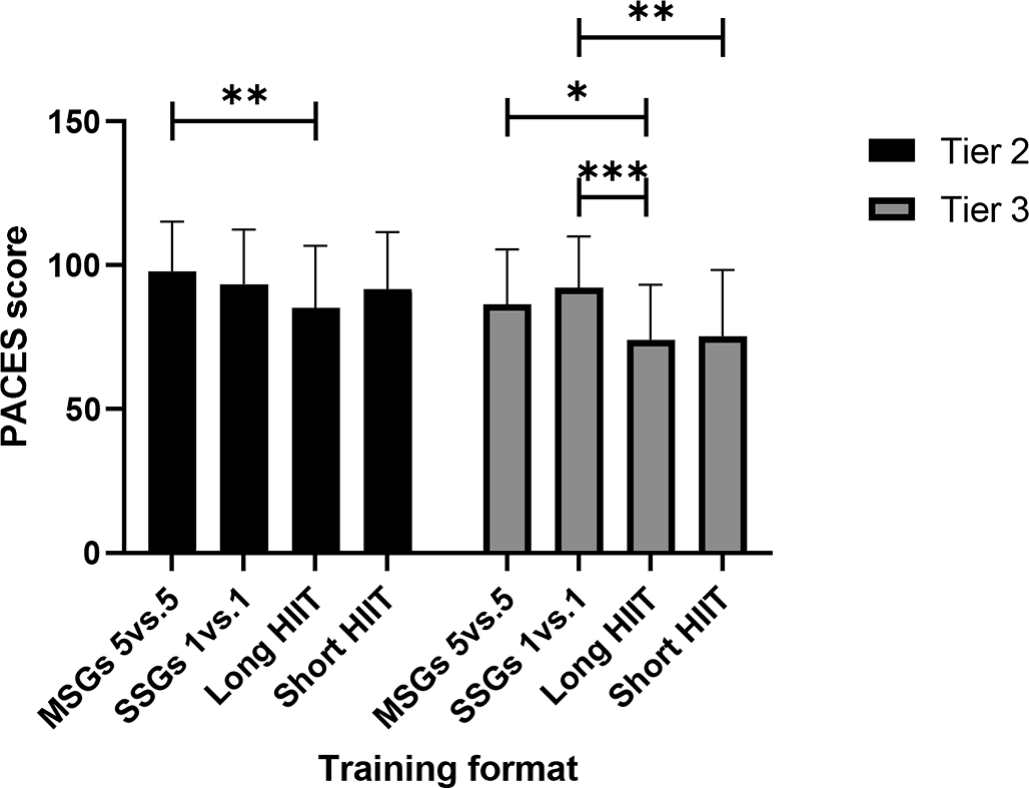
PACES score with simple main effect for differences between training formats within each competitive level. *Denotes significance at p < 0.05; **Denotes significance at p < 0.01; ***Denotes significance at p < 0.001. Note: SSGs = Small-Sided Games; HIIT = High-Intensity Interval Training; MSGs = Medium Sided-Games

Figure 2 illustrates the simple main effects of differences between training formats within each competitive level. For Tier 2, the training format significantly influenced PACES scores [F (3, 108) = 7.96, p < .001, $\eta_{\text{p}}^{2}$ = .181]. Pairwise comparisons revealed no significant differences between: 5 v 5 MSGs and 1 v 1 SSGs (M difference = 4.51, SE = 2.53, p = .494), 5 v 5 MSGs and short HIIT (M difference = 6.16, SE = 2.21, p = .051), 1 v 1 SSGs and long HIIT (M difference = 8.11, SE = 3.18, p = .090), 1 v 1 SSGs and short HIIT (M difference = 1.65, SE = 2.10, p = 1.000), short HIIT and long HIIT (M difference = 6.46, SE = 2.52, p = .088). However, PACES scores were statistically significantly lower for long HIIT than 5 v 5 MSGs (M difference = 12.62, SE = 3.01, p = .001).

For Tier 3, the training format also significantly affected PACES scores [F (3, 117) = 11.24, p < .001, $\eta_{\text{p}}^{2}$ = .224]. No significant differences were found between: 1 v 1 SSGs and 5 v 5 MSGs (M difference = 5.73, SE = 3.16, p = .469), 5 v 5 MSGs and short HIIT (M difference = 11.08, SE = 4.52, p = .113), short HIIT and long HIIT (M difference = 1.28, SE = 3.46, p = 1.000). However, PACES scores were statistically significantly lower for long HIIT compared to 5 v 5 MSGs (M difference = 12.35, SE = 3.70, p = .011), for long HIIT compared to 1 v 1 SSGs (M difference = 18.08, SE = 2.88, p < .001), for short HIIT compared to 1 v 1 SSGs (M difference = 16.80, SE = 4.17, p = .002).

### HRmean

There were no outliers, as assessed by examination of studentized residuals for values greater than ± 3. The data was normally distributed, as assessed by Kolmogorov-Smirnov’s test (p > .05). There was homogeneity of variances (p > .05) and covariances (p > .001), as assessed by Levene’s test of homogeneity of variances and Box’s M test, respectively. Mauchly’s test of sphericity indicated that the assumption of sphericity was met for the two-way interaction [χ^2^(5) = 6.37, p = .272].

The two-way mixed ANOVA showed there was no statistically significant interaction between the training format and competitive levels on HRmean [F (3, 225) = 1.49, p = .218, $\eta_{\text{p}}^{2}$ = .019]. The main effect of competitive level showed that there was a statistically significant difference in HRmean between levels [F (1, 75) = 25.07, p < .001, $\eta_{\text{p}}^{2}$ = .251]. The main effect of training formats showed a statistically significant difference in HRmean at the different formats [F (3, 225) = 46.721, p < .001, $\eta_{\text{p}}^{2}$ = .384].

**FIG. 3:**
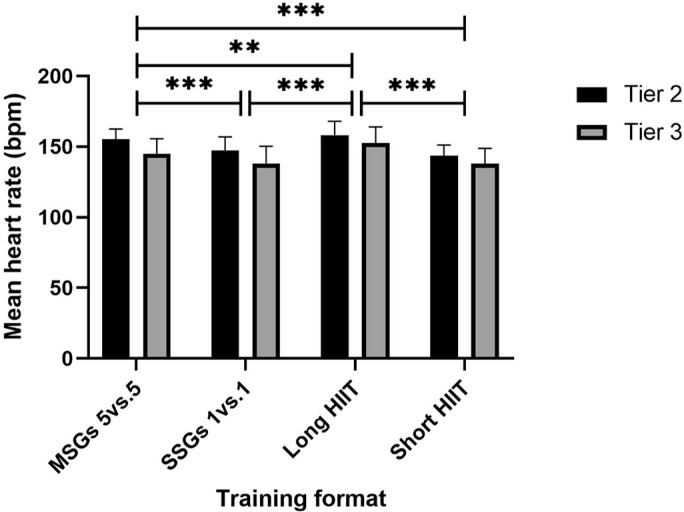
HRmean with main effect for differences between training formats. **Denotes significance at p < 0.01; ***Denotes significance at p < 0.001. Note: SSGs = Small-Sided Games; HIIT = High-Intensity Interval Training; MSGs = Medium Sided-Games; HR = Heart Rate; bpm = beats per minute.

Pairwise comparisons revealed significant differences between 5 v 5 MSGs and 1 v 1 SSGs (M difference = 7.36, SE = 1.45, p < .001), long HIIT and 5 v 5 MSGs (M difference = 5.27, SE = 1.52, p = .005), and 5 v 5 MSGs and short HIIT (M difference = 9.41, SE = 1.24, p < .001). Additionally, long HIIT differed significantly from both 1 v 1 SSGs (M difference = 12.63, SE = 1.34, p < .001) and short HIIT (M difference = 14.68, SE = 1.40, p < .001). The only non-significant comparison was between 1 v 1 SSGs and short HIIT (M difference = 2.06, SE = 1.45, p = .961). Figure 3 illustrates the main effect of HRmean across training formats.

The descriptive statistics for HRmean were as follows: 5 v 5 MSGs – 155.19 ± 7.18 bpm (Tier 2), 145.03 ± 10.51 bpm (Tier 3); 1 v 1 SSGs – 147.38 ± 9.49 bpm (Tier 2), 138.13 ± 12.15 bpm (Tier 3); Long HIIT – 158.11 ± 9.83 bpm (Tier 2), 152.65 ± 11.29 bpm (Tier 3); Short HIIT – 143.54 ± 7.59 bpm (Tier 2), 137.85 ± 10.97 bpm (Tier 3).

### RPE

There were no outliers, as assessed by examination of studentized residuals for values greater than ± 3. Although the Kolmogorov-Smirnov test indicated that some cells deviated from normality, the Normal Q-Q plots suggested that the data distribution was approximately normal. There was homogeneity of variances (p > .05) and covariances (p > .001), as assessed by Levene’s test of homogeneity of variances and Box’s M test, respectively. Mauchly’s test of sphericity indicated that the assumption of sphericity was met for the twoway interaction [χ^2^(5) = 7.43, p = .191]. There was a statistically significant interaction between the intervention and time on RPE [F (3, 225) = 3.91, p = .009, $\eta_{\text{p}}^{2}$ = .050].

There was no statistically significant difference in RPE between competitive levels for 5 v 5 MSGs [F (1, 75) = 0.58, p = .450, $\eta_{\text{p}}^{2}$ = .008], 1 v 1 SSGs [F (1, 75) = 0.002, p = .963, $\eta_{\text{p}}^{2}$ = .000], and long HIIT [F (1, 75) = 0.57, p = .452, $\eta_{\text{p}}^{2}$ = .008]. However, a statistically significant difference was observed in short HIIT [F (1, 75) = 8.28, p = .005, $\eta_{\text{p}}^{2}$ = .099], where Tier 3 players reported significantly higher RPE than Tier 2 players (M difference = 1.37, SE = 0.48, p = .005).

There was a statistically significant effect of training format on RPE for the Tier 2 [F (3, 108) = 6.74, p < .001, $\eta_{\text{p}}^{2}$ = .158]. However, pairwise comparisons showed no significant differences between long HIIT and 5 v 5 MSGs (M difference = 0.84, SE = 0.42, p = .316), 5 v 5 MSGs and short HIIT (M difference = 0.60, SE = 0.47, p = 1.000), or 1 v 1 SSGs and long HIIT (M difference = 0.16, SE = 0.37, p = 1.000). In contrast, RPE was significantly lower in 5 v 5 MSGs compared to 1 v 1 SSGs (M difference = 1.00, SE = 0.36, p = .047), in short HIIT compared to 1 v 1 SSGs (M difference = 1.60, SE = 0.45, p = .007), and in short HIIT compared to long HIIT (M difference = 1.43, SE = 0.36, p = .002).

For Tier 3 players, there was a statistically significant effect of training format on RPE [F (3, 117) = 8.24, p < .001, $\eta_{\text{p}}^{2}$ = .174]. However, no significant differences were observed between long HIIT and 1 v 1 SSGs (M difference = 0.18, SE = 0.33, p = 1.000), 1 v 1 SSGs and short HIIT (M difference = 0.25, SE = 0.34, p = 1.000), or long HIIT and short HIIT (M difference = 0.43, SE = 0.35, p = 1.000). In contrast, 5 v 5 MSGs elicited a significantly lower RPE compared to 1 v 1 SSGs (M difference = 1.40, SE = 0.33, p = .001), long HIIT (M difference = 1.58, SE = 0.36, p = .001), and short HIIT (M difference = 1.15, SE = 0.38, p = .026).

**FIG. 4:**
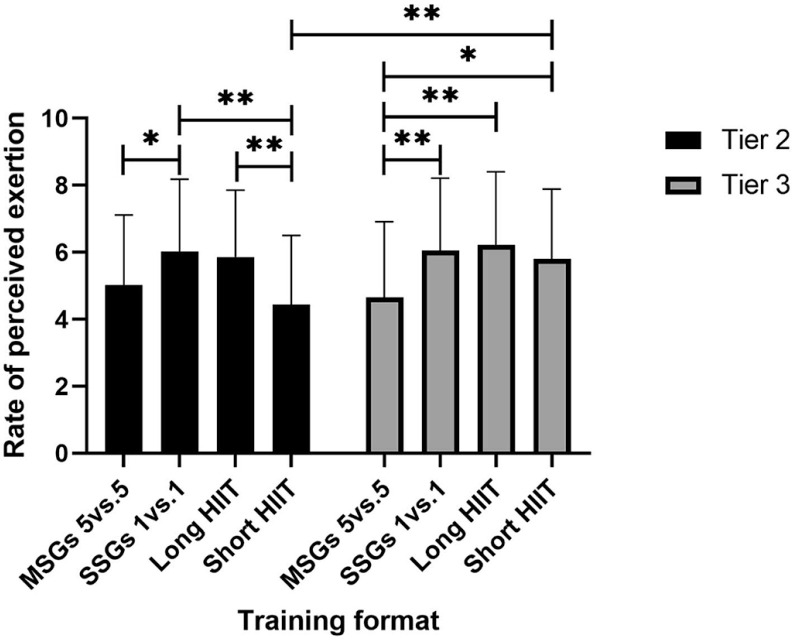
RPE score with simple main effect for differences. *Denotes significance at p < 0.05; **Denotes significance at p < 0.01; ***Denotes significance at p < 0.001. Note: SSGs = Small-Sided Games; HIIT = High-Intensity Interval Training; MSGs = Medium Sided-Games; RPE = Rate of Perceived Exertion.

Figure 4 illustrates the simple main effects of differences. RPE was measured using the Borg CR10 scale, with values expressed in points. The mean RPE scores for Tier 2 players were 5.03 ± 2.09, 6.03 ± 2.15, 5.86 ± 1.99, and 4.43 ± 2.08 points, while those for Tier 3 players were 4.65 ± 2.26, 6.05 ± 2.16, 6.23 ± 2.18, and 5.80 ± 2.09 points.

## DISCUSSION

The present study examined the PACES scores and exercise intensity differences across various SSGs and HIIT formats among soccer players in Tier 2 and Tier 3 competitive levels. The findings of the present study did not support the initial hypothesis that higher-level athletes (Tier 3) would report greater enjoyment across training formats. Specifically, the findings of the present study showed that smaller-sided formats (1 v 1) and short HIIT had the highest enjoyment levels, while Tier 2 players consistently reported higher enjoyment and physical effort than Tier 3 players.

For Tier 3 players, the 1 v 1 small-sided game format was associated with greater enjoyment levels compared to the 5 v 5 mediumsided game format. However, this pattern was not observed in Tier 2 players, where no significant difference between these formats emerged. This finding aligns with previous research showing that smaller-sided games increase ball contact, promote active participation, and consequently increase enjoyment [17, 18]. For instance, a study conducted on basketball showed that the 3 v 3 had significantly higher scores than 4 v 4 format [18]. Along the same line, another study on rugby union players found that the 7 v 7 format exhibited significantly higher PACES scores than the 10 v 10 to 12 v 12 (p = 0.007) formats [39]. However, in studies conducted on soccer players, as in the present study, no significant differences in PACES scores were found between the 3 v 3, 4 v 4, and 6 v 6 (p > 0.05) formats [31, 40]. The constrained spaces and frequent offensive and defensive transitions in SSGs offer opportunities for technical skill development, contributing to a positive training experience [13, 14]. These results suggest that coaches aiming to increase player enjoyment should integrate smaller-sided games in training programming, particularly during higher training volume periods when enjoyment may help prevent mental fatigue [41, 42].

Significant differences in enjoyment levels were observed between the HIIT and SSGs formats. In Tier 2, long HIIT sessions were significantly less enjoyable than the 5 v 5 format, with no other significant differences. In Tier 3, long HIIT had the lowest enjoyment scores, significantly lower than 5 v 5, 1 v 1, and short HIIT formats. Additionally, short HIIT was significantly less enjoyable than 1 v 1 in Tier 3. Previous studies in soccer and basketball have demonstrated that SSGs are perceived as more enjoyable than HIIT formats [7, 43]. Although one previous study [44] found no significant differences in PACES scores between the 3 v 3 SSGs format and HIIT, several other studies showed that 2 v 2, 3 v 3, and 4 v 4 SSGs formats had significantly higher PACES scores compared to HIIT formats [5, 43, 45, 46]. On the other hand, the lower enjoyment observed in the long HIIT format could be attributed to the sustained high intensity and repetitive nature of the activity, which may lead to higher perceived effort and mental fatigue [47]. It was previously shown that prolonged sets of high-intensity exercise induce greater physical discomfort and mental fatigue, which may reduce the perceived enjoyment of such protocols [48, 49]. Interestingly, short HIIT formats demonstrated moderate levels of enjoyment, suggesting that reducing the duration of high-intensity bouts might mitigate some of the negative psychological responses associated with interval training, as supported by previous studies [47, 50].

The objective and subjective measures of exercise intensity (HRmean and RPE) were higher during certain smaller-sided formats, particularly in 1 v 1 SSG formats for RPE, but not HRmean. These findings align with previous research suggesting that reducing player numbers increases movement intensity and physiological demands [51, 52]. Similarly, both Tier 2 and Tier 3 players consistently had higher RPE values in 1 v 1 SSG formats compared to the medium-sided 5 v 5 formats, reinforcing the fact that smaller-sided formats are more demanding and provide an efficient conditioning stimulus [18]. However, HRmean was higher in the 5 v 5 format compared to the 1 v 1 SSG format, indicating that exercise intensity responses may vary depending on the parameter considered. In contrast, the intensity of smaller-sided games is comparable to that of traditional HIIT sessions, with the added advantage of incorporating ball-related activities that more closely replicate match conditions [14]. As previously shown, smaller-sided games can simultaneously improve cardiorespiratory performance while improving the game’s technical and tactical aspects [53]. Particularly in competitive contexts, where balancing physical conditioning with skill development is essential, smaller-sided formats such as 1 v 1 SSG format offer a practical and effective alternative to traditional running-based HIIT formats.

Considering the differences between competitive levels, the present study found that the competitive level influenced both enjoyment and exercise intensity. Tier 2 players demonstrated higher enjoyment levels than Tier 3 players across all training formats. This finding aligns with research suggesting that psychological and physiological readiness can affect the perception of training sessions [32]. Higher-tier athletes often experience greater pressure to perform and meet strategic objectives, potentially decreasing their enjoyment levels [18]. Regarding exercise intensity, Tier 2 players exhibited higher mean across all formats, particularly during smaller-sided games and long HIIT formats. This suggests that less experienced athletes may perceive and respond to training demands differently, possibly due to variations in physical fitness, tactical knowledge, and the levels of physiological adaptations [54]. The higher RPE values in Tier 3 players during short HIIT and 1 v 1 SSGs format further reinforce the fact that training intensity seems to differ across competitive levels [55]. These findings state the importance of programming training to the specific needs of athletes at different competitive levels [56]. Ensuring high enjoyment levels, even for higher competitive level players, is critical for long-term sports adherence as part of their athletic development [57].

This study had some limitations to be acknowledged. This study used a convenience sample, which may limit the generalizability of the findings. Participants’ individual preferences for different training formats were not assessed before the study, which could have influenced their enjoyment ratings. Lastly, while HR monitoring and the RPE scale provided objective data on exercise intensity, other physiological markers (such as lactate levels or muscle fatigue) were not assessed, limiting the understanding of the full physiological impact of each training format. Future studies could benefit from incorporating additional physiological assessments for a greater understanding of training intensity and its effects on enjoyment. Despite the limitations of the study, the findings have some practical insights for soccer training programming. Incorporating smaller-sided games, particularly 1 v 1 formats, and short HIIT sessions can improve enjoyment levels and create a more positive training environment. Understanding the effects of competitive level on physical effort and enjoyment can help coaches program training to better meet the specific needs of their athletes. By combining SSGs and HIIT formats with players’ competitive levels and preferences, coaches can ensure a greater training environment that supports both physical conditioning and motivation to continue training at a great level.

## CONCLUSIONS

This study showed that SSGs and HIIT formats significantly affect enjoyment, with smaller-sided games and short HIIT formats showing the highest enjoyment levels. Tier 2 players consistently reported higher levels of enjoyment and physical effort than Tier 3 players, especially in the 5 v 5, short HIIT, and long HIIT formats. These differences suggest the need to program training sessions according to athletes’ competitive levels to maximize enjoyment and manage physical effort. For Tier 2 players, smaller-sided games and shorter HIIT sessions may improve training adherence, while Tier 3 players may benefit from longer sessions and larger formats that match their competitive demands.
